# Advanced Heart Failure Secondary to Chagas Cardiomyopathy: A Case of Successful Left Ventricular Assist Device Placement

**DOI:** 10.7759/cureus.85141

**Published:** 2025-05-31

**Authors:** Sonal Kumar, Carlos Rincon-Vazquez, Patricia Ward, Taylor E Collignon, Taisiya Tumarinson

**Affiliations:** 1 Surgery, Ross University School of Medicine, Miramar, USA; 2 Internal Medicine, Ross University School of Medicine, Miramar, USA; 3 Internal Medicine, St. George's University School of Medicine, West Indies, GRD; 4 Internal Medicine, Lake Erie College of Osteopathic Medicine, Bradenton, USA; 5 Infectious Disease, Cleveland Clinic Florida, Weston, USA

**Keywords:** acute-on-chronic heart failure, advanced heart failure, cardiogenic shock, chagas cardiomyopathy, ejection fraction, heart failure, implantable cardioverter-defibrillator, left ventricular assist device, right heart catheterization, trypanosoma cruzi

## Abstract

Chagas cardiomyopathy is a rare but critical cause of nonischemic heart failure, particularly in patients from endemic regions. We present a 48-year-old Spanish-speaking male from Guatemala with hypertension, hyperlipidemia, and heart failure with reduced left ventricular ejection fraction (LVEF, 10%) due to Chagas disease. Despite guideline-directed medical therapy and implantable cardioverter-defibrillator (ICD) placement, he developed cardiogenic shock. Right heart catheterization confirmed severely reduced cardiac output and elevated wedge pressure. He was ineligible for heart transplantation due to limited life expectancy but had no contraindications to left ventricular assist device (LVAD) placement. Following infectious disease clearance and prophylaxis for *Trypanosoma cruzi*, he successfully underwent LVAD implantation. At one-month follow-up, he showed clinical stability, improved symptoms, and adherence to medical therapy. Our case discusses the role of LVAD as a life-extending option in Chagas cardiomyopathy for non-transplant candidates, emphasizing the importance of multidisciplinary care in managing both cardiac and infectious components.

## Introduction

Chagas cardiomyopathy, the most severe and life-threatening manifestation of chronic *Trypanosoma cruzi* infection, is a leading cause of nonischemic cardiomyopathy in Latin America and an emerging global health concern [1]. With increasing global migration, it is estimated that more than six million people are infected worldwide, including more than 300,000 in the United States-many of whom are unaware of their infection status due to under-recognition in non-endemic areas. In these regions, lack of routine screening, limited awareness among healthcare providers, and asymptomatic early stages contribute to delayed or missed diagnoses, often until advanced cardiac involvement.

Chagas cardiomyopathy is pathophysiologically distinct from other cardiomyopathies, characterized by diffuse myocardial fibrosis, chronic inflammation, and autonomic dysfunction. These changes predispose patients to a spectrum of complications including ventricular arrhythmias, conduction abnormalities, thromboembolism, and progressive heart failure [2]. Diagnostic challenges are amplified by the disease’s ability to mimic other forms of cardiomyopathy, and confirmatory diagnosis requires serologic testing for *T. cruzi*, which is not routinely performed in many non-endemic countries.

Left ventricular assist devices (LVADs) have become a cornerstone in the management of end-stage heart failure, serving as either destination therapy or a bridge to transplantation. However, the use of LVADs in patients with Chagas cardiomyopathy is not well-established. Distinct clinical considerations include the impact of extensive myocardial fibrosis on device function and surgical outcomes, an elevated risk of life-threatening arrhythmias, and concerns over *T. cruzi* reactivation, particularly in immunosuppressed transplant candidates [3]. These factors necessitate a tailored, multidisciplinary approach to optimize patient outcomes.

We present a case of successful LVAD implantation in a patient with end-stage Chagas cardiomyopathy, highlighting both the feasibility of advanced mechanical support in this unique population and the clinical strategies necessary to manage its associated complexities.

## Case presentation

A 48-year-old Spanish-speaking male from Guatemala presented with advanced heart failure secondary to Chagas cardiomyopathy. His medical history was significant for hyperlipidemia, hypertension, nonischemic cardiomyopathy, and chronic heart failure with reduced ejection fraction (HFrEF). He had a left ventricular ejection fraction (LVEF) of 10%, prompting the placement of an implantable cardioverter-defibrillator (ICD), and was on Eliquis for a suspected left ventricular thrombus.

The patient first sought cardiology care two years after his initial diagnosis, reporting progressive symptoms, including dyspnea on exertion, fatigue, orthopnea, and paroxysmal nocturnal dyspnea (PND). These symptoms severely limited his exercise tolerance and were compounded by socioeconomic barriers that delayed earlier treatment. Despite the initiation of medical therapy, including dobutamine support, his condition continued to deteriorate. He experienced significant weight loss of 30 pounds and intermittent chest pain rated at 2/10. During hospitalization, cardiac MRI revealed a severely dilated left ventricle with an LVEF of 14%, though no thrombus was confirmed. An ICD was implanted, and further evaluation was undertaken to assess his candidacy for either an LVAD or an orthotopic heart transplant (OHT).

Over the following year, the patient’s condition worsened, with multiple hospitalizations for heart failure exacerbations. A transplant team excluded him from the OHT list due to his classification as New York Heart Association (NYHA) Class I. Months later, he presented to the emergency department with acute abdominal pain and worsening heart failure symptoms, including shortness of breath, orthopnea, and cardiogenic shock. He required admission to the intensive care unit, where he was managed with vasopressor support, a milrinone infusion, and diuretic therapy. Right heart catheterization demonstrated severe hemodynamic compromise, with elevated right atrial pressure, pulmonary capillary wedge pressure, and systemic vascular resistance, as well as a reduced cardiac index of 1.53 L/min/m².

A multidisciplinary team evaluated the patient’s options. Thoracic surgery determined that his life expectancy was less than two years, disqualifying him from transplantation. However, there were no contraindications to LVAD placement. Infectious disease evaluation confirmed immunity to MMR and VZV, seropositivity for *Toxoplasma*, cytomegalovirus (CMV), Epstein-Barr virus (EBV), and herpes simplex virus (HSV), and positive serology for *T. cruzi*. Strongyloides serology was pending, and TB Quantiferon was negative. The patient was cleared for LVAD implantation after receiving vaccinations for Hepatitis A, Hepatitis B, PCV20 (pneumococcal conjugate vaccine), Tdap (diphtheria, tetanus, and acellular pertussis booster vaccine), and Shingrix.

**Figure 1:**
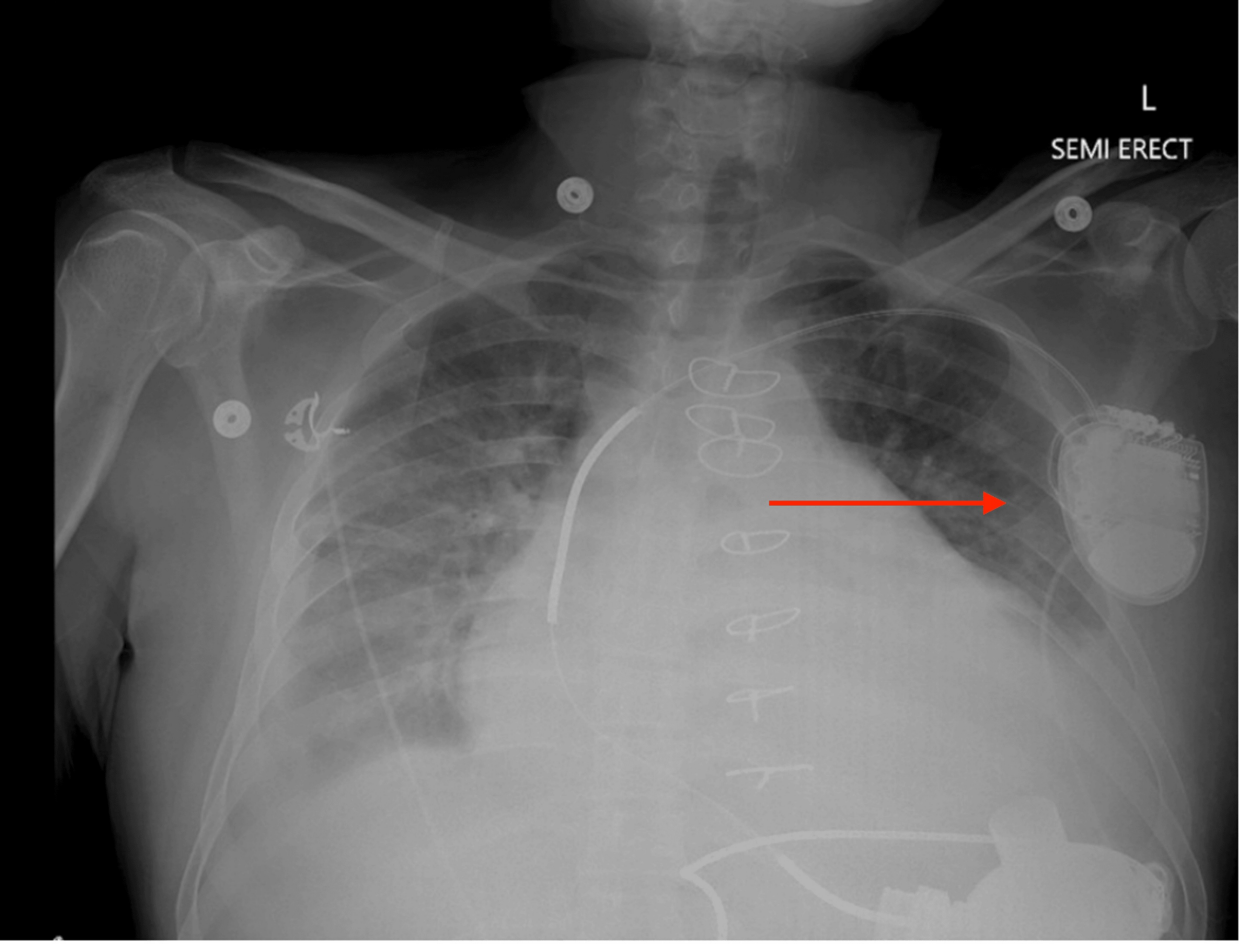
Chest X-ray demonstrating stable postoperative changes of median sternotomy and a left ventricular assist device in place (arrow), along with an automatic implantable cardioverter-defibrillator on the left side

A transesophageal echocardiogram confirmed an LVEF of 10%. The patient underwent successful LVAD placement, which served as a bridge to transplantation or destination therapy (Figure 1). At follow-up, he demonstrated clinical stability, improved functional capacity, and stable laboratory values. He was advised to adhere to LVAD speed settings, maintain his medication regimen, and observe sternal precautions.

This case highlights the importance of multidisciplinary care in managing advanced heart failure secondary to Chagas cardiomyopathy. LVAD implantation proved to be a life-saving intervention in this patient, emphasizing its feasibility in this unique population. Further research is warranted to refine management strategies for Chagas-related heart failure and to better define the role of LVADs in these cases.

## Discussion

This case highlights the challenges and complexities of managing advanced heart failure secondary to Chagas cardiomyopathy, particularly in patients with significant comorbidities and limited healthcare access [4]. Chagas cardiomyopathy is a unique form of nonischemic cardiomyopathy caused by chronic *T. cruzi *infection, which is characterized by extensive myocardial fibrosis, chronic inflammation, and conduction system abnormalities. These pathophysiological hallmarks not only drive the progressive nature of the disease but also pose significant challenges for advanced heart failure interventions, including LVAD therapy [5].

The patient presented with end-stage heart failure and severe left ventricular dysfunction, reflected by an ejection fraction of 10%. His hemodynamic profile on right heart catheterization demonstrated severely reduced cardiac output and increased pulmonary capillary wedge pressure, consistent with cardiogenic shock (Society of Cardiovascular Angiography and Interventions (SCAI) Stage D). These findings, coupled with his rapid clinical deterioration, necessitated advanced circulatory support. Although heart transplantation is the definitive treatment for end-stage heart failure [6], this patient was not a transplant candidate due to his limited life expectancy. As such, LVAD implantation was pursued as a bridge-to-decision strategy, providing an opportunity to stabilize his condition while addressing the systemic complications of Chagas cardiomyopathy.

The use of LVADs in Chagas cardiomyopathy is relatively novel and presents unique challenges. Myocardial fibrosis and conduction abnormalities increase the risk of intraoperative and postoperative complications, including arrhythmias and thromboembolic events. Furthermore, chronic inflammation and fibrosis can influence LVAD placement and function, potentially complicating outcomes. In this case, multidisciplinary collaboration among cardiology, thoracic surgery, and infectious disease specialists was pivotal in addressing these challenges.

Infectious disease management played a crucial role in ensuring the patient’s safety during LVAD implantation. *T. cruzi *reactivation, a known complication of immunosuppression, necessitated thorough preoperative evaluation and prophylactic planning. The patient underwent serological testing for common pathogens, immunization against preventable infections, and close monitoring for reactivation. This proactive approach minimized the infectious risks associated with LVAD therapy and the underlying Chagas disease.

Despite the complexities, the patient tolerated LVAD implantation well, with significant improvement in functional status during follow-up. This outcome underscores the feasibility and potential benefit of LVAD therapy in selected patients with end-stage Chagas cardiomyopathy. However, further research is needed to better define patient selection criteria, optimal perioperative strategies, and long-term outcomes in this population.

This case also highlights the importance of addressing social determinants of health in managing advanced heart failure [7]. The patient’s limited access to healthcare likely delayed his diagnosis and initiation of guideline-directed medical therapy, contributing to his disease progression. Expanding access to care, particularly in underserved populations, is essential to improve outcomes for patients with Chagas cardiomyopathy [8].

Our case illustrates that LVAD therapy can be a viable and life-saving option for patients with end-stage Chagas cardiomyopathy who are ineligible for transplantation. A multidisciplinary approach that incorporates tailored surgical strategies, infectious disease management, and attention to social factors is essential for optimizing outcomes in this challenging patient population.

## Conclusions

This case highlights the complexities of managing advanced heart failure secondary to Chagas cardiomyopathy, particularly in patients with extensive comorbidities and limited access to healthcare. The successful implantation of an LVAD in this patient demonstrates the potential for LVAD therapy as a treatment option for patients with end-stage heart failure who are not candidates for heart transplantation. However, given the unique challenges posed by myocardial fibrosis, arrhythmias, and the risk of *T. cruzi *reactivation, a multidisciplinary approach involving cardiology, thoracic surgery, and infectious disease specialists is crucial to optimize patient outcomes. While this case supports the feasibility of LVAD therapy, the findings are limited by the single-case nature and short follow-up, and caution is warranted in generalizing these results to a broader patient population. Further research with larger cohorts and longer follow-ups is needed to better define the role of LVADs in Chagas cardiomyopathy and refine management strategies for this patient group.
